# Perioperative risk factors in patients with a femoral neck fracture – influence of 25-hydroxyvitamin D and C-reactive protein on postoperative medical complications and 1-year mortality

**DOI:** 10.1186/s12891-016-0906-1

**Published:** 2016-02-01

**Authors:** Johannes KM Fakler, Antonia Grafe, Jamila Dinger, Christoph Josten, Gabriela Aust

**Affiliations:** Department of Orthopedics, Trauma and Plastic Surgery, University Hospital Leipzig AöR, Liebigstr. 20, 04103 Leipzig, Germany; Research Laboratories of the Department of Orthopedics, Trauma and Plastic Surgery, University Hospital of Leipzig AöR, Liebigstr. 20, Leipzig, 04103 Germany

**Keywords:** Hip fracture, 25-hydroxyvitamin D, C-reactive protein, Mortality, Morbidity

## Abstract

**Background:**

This study examined the association of 25-hydroxyvitamin D (25(OH)D) and C-reactive protein (CRP) with postoperative medical complications and one year mortality of elderly patients sustaining a low-energy cervical hip fracture scheduled for surgery. We hypothesized that vitamin D deficiency and CRP in these patients might be associated with an increased 1-year mortality.

**Methods:**

The prospective single-center cohort study included 209 patients with a low-energy medial femoral neck fracture; 164 women aged over 50 years and 45 men aged over 60 years. Referring to 1-year mortality and postoperative medical complications multiple logistic regression analysis including 10 co-variables (age, sex, BMI, ASA, creatinine, CRP, leukocytes hemoglobin, 25(OH)D, vitamin D supplementation at follow-up) was performed.

**Results:**

Vitamin D deficiency was prevalent in 87 % of all patients. In patients with severe (<10 ng/ml) and moderate (10–20 ng/ml) vitamin D deficiency one year mortality was 29 % and 13 %, respectively, compared to 9 % in patients with > 20 ng/ml 25(OH)D levels (*p* =0.027). Patients with a mild (CRP 10–39.9 mg/l) or active inflammatory response (CRP ≥ 40 mg/l) showed a higher one year mortality of 33 % and 40 % compared to 16 % in patients with no (CRP < 10 mg/l) inflammatory response (*p* = 0.002). Multiple logistic regression analysis identified CRP (OR 1.01, 95 % CI 1.00-1.02; *p* = 0.007), but not 25(OH)D (OR 0.97, 95 % CI 0.89-1.05; *p* = 0.425) as an independent predictor for one year mortality. 20 % of patients suffered in-hospital postoperative medical complications (i.e. pneumonia, thromboembolic events, etc.). 25(OH)D (OR 0.89, 95 % CI 0.81–0.97; *p* = 0.010), but not CRP (OR 1.01, 95 % CI 1.00-1.02; *p* = 0.139), was identified as an independent risk factor.

**Conclusion:**

In elderly patients with low-energy cervical hip fracture, 25(OH)D is independently associated with postoperative medical complications and CRP is an independent predictor of one year mortality.

## Background

Hip fractures are incisive events for elderly people. Overall complication rates after surgery for hip fracture add up to almost 50 % [1]. Approximately half of hip fracture patients fall at least once within 12 months [2] after surgery resulting in additional fractures in 10-20 % of these patients [3]. Every third hip fracture patient needs to be readmitted due to postoperative morbidity for any reason within one year after surgery [2, 4]. Moreover, up to 30 % of the patients with a hip fracture die within the first year [5] which is an excess mortality of 8-18 % at one year compared to matching cohorts without a hip fracture [6].

Identification of risk factors offers the chance to determine patients prone to postoperative morbidity and mortality in the first year after hip fractures. Patient specific factors as age, gender, general health status and comorbidities, i.e. cardiac disease and dementia, have been identified as potential risk factors [7–9]. Apart from these factors, routine laboratory parameters can predict mortality. Low hemoglobin, total leukocyte count and albumin, as well as high creatinine and parathyroid hormone (PTH) levels are associated with a higher probability of death after one year in hip fracture patients [10].

The potential influence of serum 25-hydroxyvitamin D (25(OH)D on mortality is discussed controversially. Many studies demonstrated an independent inverse relationship between 25(OH)D and mortality [11–13] while other studies did not [14, 15]. In hip fracture patients no independent association between 25(OH)D and hospital mortality or one year mortality was found [16, 17]. Only limited information is available on 25(OH)D as a potential risk factor of postoperative medical complications [16]. To our knowledge, no information is available on CRP as a potential predictor of one year mortality in hip fracture patients but there is data showing no relationship between CRP and mortality at 3 month postoperatively [18].

We hypothesized that low levels of 25(OH)D and elevated levels of CRP are associated with increased postoperative medical complications and one year mortality of elderly patients that sustained a low-energy hip fracture.

## Methods

### Ethical statement and patients

The study was approved by the local Ethics Committee at the University of Leipzig (243-11-11072011). Written informed consent was obtained from all patients or their legal representative.

For this prospective single-center cohort study, all patients were enrolled from January 2011 to March 2014. Female patients older than 50 years and male patients older than 60 years that suffered a low-energy, medial femoral neck fracture and were scheduled for surgery, were included. Patients presenting with a pathologic fracture or sustaining a high-energy trauma were excluded. 209 patients were enrolled, with 164 females accounting for 79 %. Median (interquartile range; IQR) age of the patients was 81 (73-87) years. Comorbidities were assessed according to the American Society of Anaesthesiologists (ASA) score [19].

### Surgical treatment

All patients received general anesthesia and were treated operatively. Patients with undisplaced or minimal displaced fractures (Garden I/II) were generally scheduled for internal fixation with a dynamic hip screw (33 %) (DHS; DePuy-Synthes, West Chester, USA). In displaced fractures (Garden III/IV) a bipolar hemiprosthesis (62 %) (DePuy, Warsaw, USA) was implanted. Patients with a displaced fracture and concomitant advanced coxarthrosis or a high functional demand received total hip arthroplasty (5 %) (DePuy, Warsaw, USA). Final decision on implant choice also considered was individual patient characteristics.

### Follow-up

Postoperative medical complications that necessitated specific therapeutic intervention were recorded during the stay in hospital and classified into cardiovascular, thromboembolic, pulmonary infection, extra-pulmonary infection and others (i.e. delirium).

At 6 and 12 months after surgery patients or their authorized representative and, if necessary, their general practitioner, were contacted by phone and asked about walking ability and pain in the concerned hip according to the Merle d’Aubigné score [20]. Additionally, complications and medication for osteoporosis treatment were documented. No patient was lost during follow-up at 12 months. Two patients (1 %) refused to answer the questions of the protocol but were still alive at one year postoperatively. General postoperative complications were recorded, if subsequent specific medical therapy was necessary.

### Blood samples and biochemical methods

Blood samples for routine laboratory parameters, in particular creatinine, CRP, hemoglobin and total leukocyte count were drawn immediately after admission to the emergency room. An additional fasting blood sample for analysis of circulating 25(OH)D was taken between 7.00 and 10.00 am preoperatively in 70 % and postoperatively at day 3 to 5 in 30 % of the patients. In 7 patients both pre- and postoperative levels of 25(OH)D were analyzed demonstrating no significant difference (preoperative 8.4 [6.3 – 17.7], postoperative 8.5 [7.4 - 14.5] mg/ml 25(OH)D; *p* = 0.156). Serum 25(OH)D was determined with the LIAISON^®^ 25-OH Vitamin D assay (DiaSorin, Stillwater, MN, USA). The degree of vitamin D deficiency was classified as severe (<10 ng/ml), moderate (10–19.9 ng/ml), insufficient (20–29.9 ng/ml) and normal (≥30 ng/ml) [21]. Recommendation for oral Vitamin D (1.000 IE/day) and calcium supplementation was given in the discharge letter for all patients. According to Clyne and Olshaker [22] CRP levels were divided into three groups: no inflammatory response (<10 mg/l), mild (10–39.9 mg/l) and active inflammatory response (≥40 mg/l).

### Statistical analysis

Sample size calculation for a logistic regression model was performed with the statistical program G-Power (University of Düsseldorf, Germany). We estimated an increased mortality risk of at least 60 % in subjects with vitamin D deficiency based on observational studies [11] and own experience. With an assumed α-error of 5 % and a power of 80 % a total necessary sample size of 182 patients was calculated. Post hoc power analysis confirmed that targeted sample sizes provided 91 % power (α = 0.05, β = 0.91) to detect a significant difference in mortality rates of patients with severe vitamin D deficiency compared to all other patients.

Normally distributed parameters were given as mean and standard deviation (SD), for non-normally distributed parameters, median and the interquartile range [25th–75th percentile] were used. The following statistical tests were applied: Kaplan-Meier survival analysis, log rank (Mantel-Cox), Spearman correlation, and Wilcoxon test. Binary multivariable regression analysis was performed with stepwise selection and inclusion of ten co-variables: age, sex, BMI, ASA, vitamin D supplementation at hemoglobin, total leukocyte count, creatinine, CRP and 25(OH)D. For 1-year mortality rates vitamin D supplementation at follow-up and for postoperative medical complications vitamin D supplementation at admission was added as a co-variable. All statistical computations were performed using SPSS version 20.0 (Chicago, IL, USA). *P* values less than 5 % were considered as significant.

## Results

**Fig. 1:**
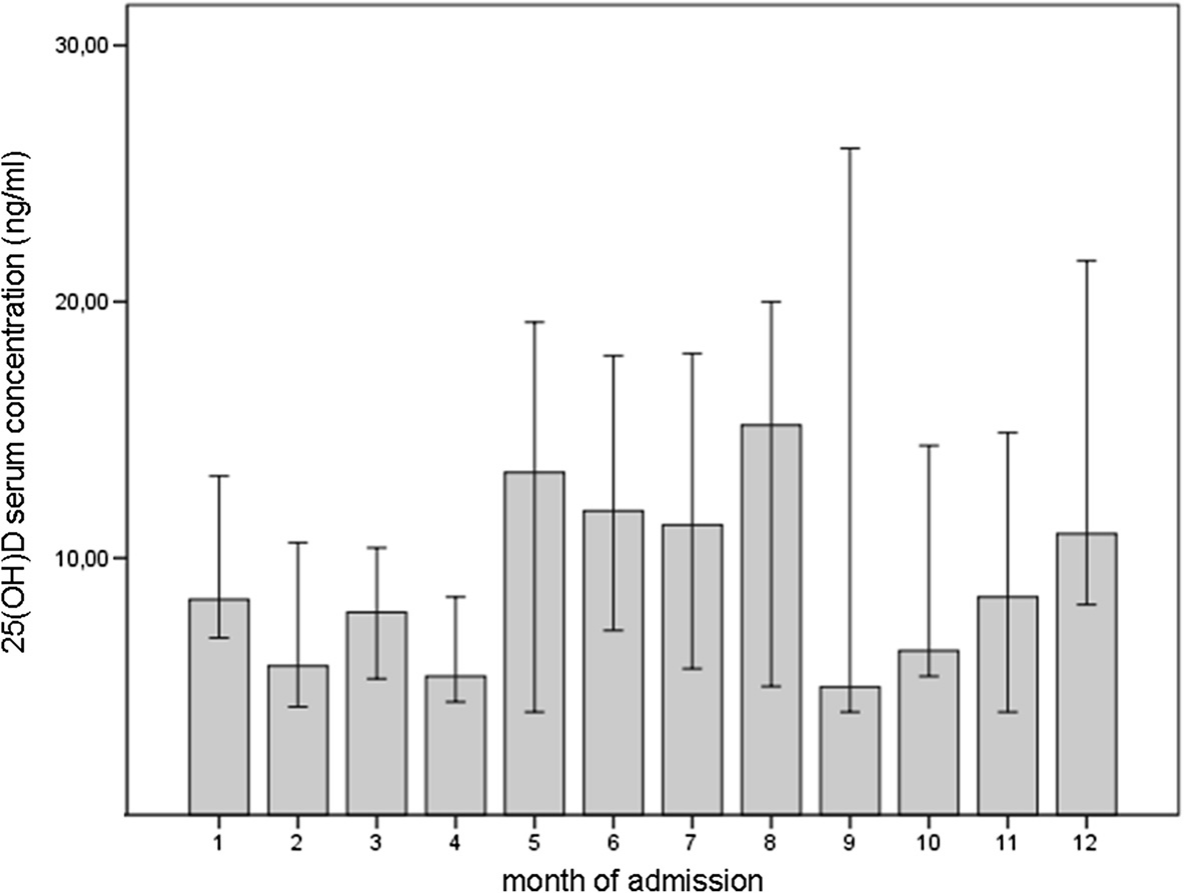
Monthly variation of 25(OH) D levels (median, 95 % CI)

At admission to hospital, normal and insufficient 25(OH)D levels were present in only 1 % and 12 % of the patients, respectively. Moderate and severe 25(OH)D deficiency were prevalent in 28 % and 59 % of the patients (Table 1). In the annual period the variation of 25(OH)D in the study cohort was low and variations of monthly median values did not exceed 15 ng/ml (Fig. 1). From February to April and September to November 25(OH)D monthly median levels of all patients were below 10 ng/ml. At admission to hospital, only 10 % of the patients supplemented vitamin D. This rate increased to 19 % after discharge from hospital.

**Table 1:** Baseline characteristics and postoperative results of all patients

Age (years)	81 (73–87)
sex (female)	79 %
BMI	25 (22–28)
ASA	I: 2 % II: 27 % III: 69 % IV: 2 %
creatinine (μmol/l)	73 (60–97)
haemoglobin (mmol/l)	7.9 (7.2-8.6)
leukocytes (exp9/l)	10.2 (8.0-13-3)
CRP (mg/l)	5.3 (1.8-15.4)
25(OH)D (ng/ml)	8.4 (5.1-14.5)
Postoperative medical complications (total)	20 %
cardiovascular	6 %
thromboembolic	1 %
pulmonary infection	3 %
extra-pulmonary infections	4 %
other (i.e. delirium)	6 %
30-day mortality	13 %
1-year mortality	23 %

Values given as median (IQR) or as percentage

We found correlations of 25(OH)D with CRP (*r* = −0.245, *p* = 0.001), age (*r* = −0.278, *p* < 0.001), and ASA score (*r* = −0.267, *p* < 0.001). CRP only correlated weakly with the ASA score (*r* = 0.155, *p* = 0.026) (Table 2).

**Table 2:** Correlation of age, ASA-classification, CRP and 25(OH)D

		Age (years)	ASA	CRP (mg/l)	25 (OH)D (ng/ml)
Age (years)	correlation (rho *r*)	1.000	0.322	0.34	−0.278
	significance (*p*)		<0.001*	0.623	<0.001*
ASA	correlation (rho *r*)	0.322	1.000	0.155	−0.267
	significance (*p*)	<0.001*		0.026*	<0.001*
CRP (mg/l)	correlation (rho *r*)	0.034	0.155	1.000	−0.245
	significance (*p*)	0.623	0.026*		0.001*
25 (OH)D (ng/ml)	correlation (rho *r*)	−0.278	−0.267	−0.245	1.000
	significance (*p*)	<0.001*	<0.001*	0.001*	

*p < 0.05

**Fig. 2:**
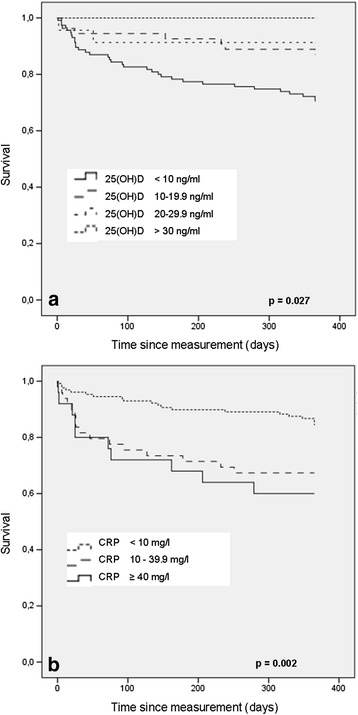
Kaplan Meier survival curves of 209 patients with hip fracture for serum 25(OH)D **a** and CRP **b**

Mortality after 1 year was 29 %, 13 % and 9 % for patients with severe and moderate 25(OH)D deficiency or insufficient 25(OH)D levels, respectively. All patients with normal 25(OH)D levels survived the first year (Fig. 2a). The inverse relationship between 25(OH)D and mortality was significant (*p* = 0.027). Dividing 25(OH)D levels into quartiles showed similar results with a mortality rate of 6 % in the highest, 23 % and 24 % in the intermediate quartiles and 34 % in the lowest quartile (*p* = 0.014). Multivariable logistic regression analysis adjusted for age, sex, BMI, ASA score and potential confounders as creatinine, CRP, haemoglobin, total leukocyte count, and vitamin D supplementation at follow-up revealed no independent association of 25(OH)D and mortality (OR 0.97, 95%CI 0.89-1.05, *p* = 0.425) (Table 3).

**Table 3:** Unadjusted and adjusted risk factors influencing one year mortality in hip fracture patients in uni- and multivariable logistic regression models

	Unadjusted OR (95 % CI)	*p*-value	Adjusted OR (95 % CI)	*p*-value
age	1.11 (1.06-1.16)	<0.001^*^	1.10 (1.04-1.18)	0.002^*^
sex	1.81 (0.75-4.36)	0.187	2.05 (0.57-7.43)	0.273
BMI	1.02 (0.96-1.08)	0.569	1.03 (0.96-1.11)	0.412
ASA	7.83 (2.76-22.21)	<0.001^*^	3.59 (1.04-12.33)	0.043^*^
creatinine	1.01 (1.00-1.01)	0.105	1.01 (1.00-1.02)	0.022^*^
CRP	1.01 (1.01-1.02)	<0.001^*^	1.01 (1.00-1.02)	0.007^*^
leukocytes	1.04 (0.97-1.11)	0.268	1.02 (0.98-1.06)	0.375
hemoglobine	0.81 (0.59-1.09)	0.166	0.99 (0.68-1.43)	0.946
vitamin D at follow-up	0.43 (0.16-1.16)	0.096	0.34 (0.07-1.54)	0.160
25(OH)D	0.90 (0.84-0.96)	0.003^*^	0.97 (0.89-1.05)	0.425

Age, sex, BMI, ASA and blood test parameters at admission, vitamin D supplementation at follow-up (*OR* odds ratio, *CI* confidence interval). R2 (Nagelkerke) = 0.399. ^*^ p < 0.05.

No inflammatory response (CRP < 10 mg/l) was seen in 64 % patients, while a mild and active response was prevalent in 24 % and 12 % of the patients (Table 1). Kaplan-Meier survival curves demonstrate a significantly higher mortality rate of up to 40 % for patients with CRP levels ≥ 10 mg/l (*p* = 0.002) (Fig. 2b). Logistic regression analysis adjusted for age, sex, BMI, ASA score and additional potential confounders as creatinine, 25(OH)D, haemoglobin, total leukocyte count, and vitamin D supplementation at follow-up revealed a significant independent association with mortality (OR = 1.014, 95 % CI = 1.00-1.02, *p* = 0.007) (Table 3).

Postoperative medical complications warranting specific therapeutic intervention were seen in 20 % of all patients. Cardiovascular and thromboembolic events accounted for 30 % and 5 %, non-surgical infections for 37 % and miscellaneous events (i.e. delirium) for 28 % of the complications (Table 1). Low 25(OH)D (OR 0.88, 95%CI 0.81-0.95, *p* = 0.002), but not high CRP (OR 1.01, 95%CI 1.00-1.02, *p* = 0.300) correlated with higher rates of postoperative medical complications in an univariable regression analysis. The multivariable model corrected for age, sex, BMI, ASA score and additional potential confounders as creatinine, CRP, haemoglobin, total leukocyte count, and vitamin D supplementation at admission confirmed 25(OH)D (OR 0.89, 95%CI 0.81-0.97, *p* = 0.010) as an independent predictor of postoperative medical complications, whereas no association was seen for CRP (OR 1.01, 95 % CI 1.00-1.02, *p* = 0.139) (Table 4).

**Table 4:** Unadjusted and adjusted risk factors influencing postoperative medical compliactions in hip fracture patients in uni- and multivariable logistic regression models

	Unadjusted OR (95 % CI)	*p*-value	Adjusted OR (95 % CI)	*p*-value
age	1.07 (1.03 – 1.11)	0.002^*^	1.04 (0.99 – 1.10)	0.137
sex	1.05 (0.46 – 2.38)	0.914	0.72 (0.23 – 2.21)	0.563
BMI	0.97 (0.90 – 1.04)	0.360	0.98 (0.90 – 1.06)	0.564
ASA	5.61 (2.15 – 14.63)	<0.001^*^	2.84 (0.92 – 8.84)	0.071
creatinine	1.01 (1.00 – 1.02)	0.490	1.02 (1.01 – 1.03)	0.005^*^
CRP	1.01 (1.00 – 1.02)	0.300	1.01 (1.00 – 1.02)	0.139
leukocytes	1.02 (0.99 – 1.05)	0.250	1.02 (0.99 – 1.04)	0.315
hemoglobine	0.82 (0.60 – 1.12)	0.211	1.07 (0.75 – 1.53)	0.709
vitamin D at admission	1.63 (0.59 – 4.49)	0.340	5.85 (1.03 – 33.23)	0.046^*^
25(OH)D	0.88 (0.81 – 0.95)	0.002^*^	0.89 (0.81 – 0.97)	0.010^*^

Age, sex, BMI, ASA and blood test parameters and vitamin D supplementation at admission (*OR* odds ratio, *CI* confidence interval). R2 (Nagelkerke) = 0.321

* p < 0.05

## Discussion

In our study cohort of hip fracture patients, a high prevalence of vitamin D deficiency was evident. 25(OH)D and one year mortality showed a significant association, however 25(OH)D was not confirmed as an independent predictor. Our results differ in part with a study of Madsen et al. [17] of 562 hip fracture patients in which 25(OH)D and mortality were not associated at all one year after surgery. Interestingly, a significant association of 25(OH)D and one year mortality was demonstrated in matched controls, although 25(OH)D levels did not differ between both groups. Notably, in the Madsen study, median 25(OH)D levels of all patients were two times higher (19.8 ng/ml), i.e. closer to the insufficient range than the deficient range, as was the case in our study cohort (10.8 ng/ml). No information on vitamin D medication of the patients was given which could explain the substantial difference of 25(OH)D levels to our study. In our patients, oral supplementation of vitamin D was recorded in 19 % one year after surgery compared to 10 % preoperatively. Consequently, this low vitamin D substitution rate suggests low, but stable levels of 25(OH)D within the observation period, because monthly variations of 25(OH)D were small, not exceeding 15 ng/ml. Our assumption is confirmed by a large population based cohort study demonstrating that baseline 25(OH)D differed marginally over 5 years [12].

Nevertheless, 25(OH)D was not an independent predictor of one year mortality in our study. Confounding factors potentially influenced 25(OH)D levels and may explain this result. Systemic inflammation has been reported to lower circulating 25(OH)D [23]. Consequently, patients with a marked inflammation might be falsely identified as vitamin D deficient. In fact, our study demonstrated an inverse association between 25(OH)D and CRP, an established indicator of systemic inflammation. However, exclusion of CRP in the multivariable regression model did not alter the role of 25(OH)D, demonstrating no independent association with one year mortality. Postoperative fluid shifts also represent a potential confounder of 25(OH)D measurements [24]. In our study, most of the blood samples for analysis of 25(OH)D were taken preoperatively. Additionally, pre- and postoperative analysis of 25(OH)D samples in the same patients did not differ significantly in our study. The association of 25(OH)D with age as well as with the ASA score which both are independent predictors of one year mortality, indicates that 25(OH)D might be regarded as a marker of general health status and thus explain its role as an overall, but not independent predictor of one year mortality. This is supported by interventional trails that lack clear evidence of 25(OH)D being a causal factor of increased mortality. A recent randomized double-blind, placebo-controlled trail, showed that supplementation of vitamin D in critically ill patients failed to reduce hospital or 6 months mortality [25].

In contrast to one year mortality, we identified 25(OH)D at admission to hospital as an independent predictor of postoperative medical complications which is in line with the majority of surgical outcome studies [26]. This result is also confirmed by several studies exhibiting an association between vitamin D deficiency and increased susceptibility to infections [27, 28]. Indeed, a meta-analysis identified vitamin D deficiency as a potential risk factor for increased infection rates and sepsis in critically ill patients [29]. Vitamin D deficiency may also be a potential cardiovascular risk factor and cause myocardial structural changes [30]. However, recent randomized double-blind, placebo-controlled trails did not confirm that supplementation of vitamin D had any beneficial effects on cardiovascular risk factors [31, 32]. In hip fracture patients, Fisher et al. [16] did not find an association between low 25(OH)D levels and postoperative complications in a study which did not consider medical complications apart from cardiac incidents.

Our study revealed that an elevated CRP at admission to hospital is an independent risk factor for one year mortality, but not postoperative medical complications. Accordingly, in a longitudinal study of 1044 elderly women aged 75 years and older at inclusion elevated levels of CRP predict higher mortality [33]. Here, in addition to CRP, age, ASA score and creatinine were identified as independent predictors of one year mortality. This is in accordance to other authors demonstrating that age and renal dysfunction independently are associated with increased 3 months [18] and 12 months [34] mortality, respectively. However, Mosfeldt et al. [18] found no association of CRP and mortality after 3 months in hip fracture patients, although CRP tended to be higher in deceased patients.

Our study is limited by its design which does not allow a causal conclusion. Furthermore, blood samples for 25(OH)D analysis were taken at different time points, although pre- and postoperative 25(OH)D levels of the same patients did not vary. Despite a priori and post hoc power analysis indicated sufficient power of this study, the number of patients might be too small to identify a potentially independent association of 25(OH)D with 1-year mortality. In addition, albumin und PTH, two potential confounding factors influencing mortality and morbidity [9], were not determined, thus impeding the interpretation of results. Also, potential clinical confounders as functional and cognitive aspects were not addressed [7–9].

## Conclusion

In conclusion, this study demonstrates that preoperatively elevated CRP levels are independently associated with increased one year mortality and 25(OH)D is an independent predictor of postoperative in-hospital medical complications. These biochemical parameters might help to identify elderly patients with a femoral neck fracture being at risk for postoperative complications or death within one year after surgery.

## Abbreviations

25(OH)D: 25-hydroxyvitamin D
ASA: American Society of Anaesthesiologists
BMI: Body mass index
CRP: C-reactive protein

## References

[1] Fields AC, Dieterich JD, Buterbaugh K, Moucha CS (2015). Short-term complications in hip fracture surgery using spinal versus general anaesthesia. Injury.

[2] Miller RR, Ballew SH, Shardell MD, Hicks GE, Hawkes WG, Resnick B (2009). Repeat falls and the recovery of social participation in the year post-hip fracture. Age Ageing..

[3] Bischoff-Ferrari HA, Dawson-Hughes B, Platz A, Orav EJ, Stählin HB, Willett WC (2010). Effect of high-dosage cholecalciferol and extended physiotherapy on complications after hip fracture: a randomized controlled trial. Arch Intern Med..

[4] Teixeira A, Trinquart L, Raphael M, Bastianic T, Chatellier G, Holstein J (2009). Outcomes in older patients after surgical treatment for hip fracture: a new approach to characterise the link between readmissions and the surgical stay. Age Ageing.

[5] Graham J, Bowen TR, Strohecker KA, Irgit K, Smith WR (2014). Reducing mortality in hip fracture patients using a perioperative approach and "Patient- Centered Medical Home" model: a prospective cohort study. Patient Saf Surg.

[6] Haentjens P, Magaziner J, Colón-Emeric CS, Vanderschueren D, Milisen K, Velkeniers B (2010). Meta-analysis: excess mortality after hip fracture among older women and men. Ann Intern Med..

[7] Diamantopoulos AP, Hoff M, Hochberg M, Haugeberg G (2013). Predictors of short- and long-term mortality in males and females with hip fracture - a prospective observational cohort study. PLoS One.

[8] Smith T, Pelpola K, Ball M, Ong A, Myint PK (2014). Pre-operative indicators for mortality following hip fracture surgery: a systematic review and meta-analysis. Age Ageing.

[9] Hu F, Jiang C, Shen J, Tang P, Wang Y (2012). Preoperative predictors for mortality following hip fracture surgery: a systematic review and meta-analysis. Injury.

[10] Laulund AS, Lauritzen JB, Duus BR, Mosfeldt M, Jørgensen HL (2012). Routine blood tests as predictors of mortality in hip fracture patients. Injury.

[11] Pilz S, Dobnig H, Nijpels G, Heine RJ, Stehouwer CD, Snijder MB (2009). Vitamin D and mortality in older men and women. Clin Endocrinol (Oxf).

[12] Schöttker B, Haug U, Schomburg L, Köhrle J, Perna L, Müller H (2013). Strong associations of 25-hydroxyvitamin D concentrations with all-cause, cardiovascular, cancer, and respiratory disease mortality in a large cohort study. Am J Clin Nutr..

[13] Amrein K, Zajic P, Schnedl C, Waltensdorfer A, Fruhwald S, Holl A (2014). Vitamin D status and its association with season, hospital and sepsis mortality in critical illness. Crit Care..

[14] Eaton CB, Young A, Allison MA, Robinson J, Martin LW, Kuller LH (2011). Prospective association of vitamin D concentrations with mortality in postmenopausal women: results from the Women’s Health Initiative (WHI). Am J Clin Nutr..

[15] Formiga F, Ferrer A, Megido MJ, Boix L, Contra A, Pujol R (2014). Low serum vitamin D is not associated with an increase in mortality in oldest old subjects: the Octabaix three-year follow-up study. Gerontology..

[16] Fisher A, Goh S, Srikusalanukul W, Davis M (2009). Elevated serum PTH is independently associated with poor outcomes in older patients with hip fracture and vitamin D inadequacy. Calcif Tissue Int.

[17] Madsen CM, Jørgensen HL, Lind B, Ogarrio HW, Riis T, Schwarz P (2012). Secondary hyperparathyroidism and mortality in hip fracture patients compared to a control group from general practice. Injury..

[18] Mosfeldt M, Pedersen OB, Riis T, Worm HO (2012). Mark Sv, Jorgensen HL, et al. Value of routine blood tests for prediction of mortality risk in hip fracture patients. Acta Orthop.

[19] Dripps RD (1963). New Classification of Physical Status. Anesthesiology.

[20] Merle d’Aubigné R, Postel M (1954). Functional results of hip arthroplasty with acrylic prosthesis. J Bone Joint Surg [Am].

[21] Holick MF (2007). Vitamin D deficiency. N Engl J Med.

[22] Clyne B, Olshaker S (1999). The C-Reactive Protein. Clin Lab Emerg Med.

[23] Ghashut RA, Talwar D, Kinsella J, Duncan A, McMillan DC (2014). The effect of the systemic inflammatory response on plasma vitamin 25 (OH) D concentrations adjusted for albumin. PLoS One.

[24] Krishnan A, Ochola J, Mundy J, et al. Acute fluid shifts influence the assessment of serum vitamin D status in critically ill patients. Crit Care 2010; 14: R216. doi: 10.1186/cc9341. Epub 2010 Nov 26 10.1186/cc9341 PMC3219984 21110839

[25] Amrein K, Schnedl C, Holl A, Riedl R, Christopher KB, Pachler C (2014). Effect of high-dose vitamin D3 on hospital length of stay in critically ill patients with vitamin D deficiency: the VITdAL-ICU randomized clinical trial. JAMA..

[26] Sahay T, Ananthakrishnan AN (2014). Vitamin D deficiency is associated with community-acquired clostridium difficile infection: a case–control study. BMC Infect Dis.

[27] Quraishi SA, Bittner EA, Christopher KB, Camargo CA (2013). Vitamin D status and community-acquired pneumonia: results from the third National Health and Nutrition Examination Survey. PLoS One.

[28] Iglar PJ, Hogan KJ (2015). Vitamin D status and surgical outcomes: a systematic review. Patient Saf Surg.

[29] de Haan K, Groeneveld A, de Geus H, Egal M, Struijs A (2014). Vitamin D deficiency as a risk factor for infection, sepsis and mortality in the critically ill: systematic review and meta-analysis. Crit Care..

[30] Pekkanen MP, Ukkola O, Hedberg P, Piira OP, Lepojärvi S, Lumme J, et al. Serum 25-hydroxyvitamin D is associated with major cardiovascular risk factors and cardiac structure and function in patients with coronary artery disease. Nutr Metab Cardiovasc Dis. 2015;S0939-4753(15):00048–4. Epub ahead of print. 10.1016/j.numecd.2015.02.005 25816731

[31] Wood AD, Secombes KR, Thies F, Aucott L, Black AJ, Mavroeidi A (2012). Vitamin D3 supplementation has no effect on conventional cardiovascular risk factors: a parallel-group, double-blind, placebo-controlled RCT. J Clin Endocrinol Metab..

[32] Pilz S, Gaksch M, Kienreich K, Grübler M, Verheyen N, Fahrleitner-Pammer A (2015). Effects of vitamin D on blood pressure and cardiovascular risk factors: a randomized controlled trial. Hypertension..

[33] Berglundh S, Malmgren L, Luthman H, McGuigan F, Akesson K (2015). C-reactive protein, bone loss, fracture, and mortality in elderly women: a longitudinal study in the OPRA cohort. Osteoporos Int..

[34] Khan SK, Rushton SP, Courtney M, Gray AC, Deehan DJ (2013). Elderly men with renal dysfunction are at most risk for poor outcome after neck of femur fractures. Age Aging..

